# Mechanical and Structural Properties of Polyhydroxybutyrate as Additive in Blend Material in Additive Manufacturing for Medical Applications

**DOI:** 10.3390/polym15081849

**Published:** 2023-04-12

**Authors:** Muhammad Zulhilmi Zainuddin, Ahmad Adnan Abu Bakar, Ahmad Nurhelmy Adam, Shahino Mah Abdullah, Nizam Tamchek, Muhammad Syafiq Alauddin, Mohd Muzamir Mahat, Nophadon Wiwatcharagoses, Ahmad Alforidi, Mohd Ifwat Mohd Ghazali

**Affiliations:** 1 SMART RG, Faculty of Science and Technology (FST), Universiti Sains Islam Malaysia (USIM), Nilai 71800, Malaysia; 2 Department of Physics, Faculty of Science, Universiti Putra Malaysia (UPM), Serdang 43400, Malaysia; 3 Department of Conservative Dentistry and Prosthodontics, Faculty of Dentistry, Universiti Sains Islam Malaysia, Kuala Lumpur 55100, Malaysia; 4 Faculty of Applied Sciences, Universiti Teknologi Mara, Shah Alam 40450, Selangor, Malaysia; 5 Department of Electrical and Computer Engineering, King Mongkut’s University of Technology North Bangkok (KMUTNB) 1518 Pracharat 1 Road, Bangkok 10800, Thailand; 6 Electrical Engineering Department, Taibah University, Medina 42353, Saudi Arabia; aforidi@taibahu.edu.sa

**Keywords:** additive manufacturing, stereolithography (SLA), polyurethane acrylate, polyhydroxybutyrate, medical application

## Abstract

Today, additive manufacturing (AM) is considered one of the vital tenets of the industry 4.0 revolution due to its high productivity, decentralized production and rapid prototyping. This work aims to study the mechanical and structural properties of polyhydroxybutyrate as an additive in blend materials and its potential in medical applications. PHB/PUA blend resins were formulated with 0 wt.%, 6 wt.%, 12 wt.% and 18 wt.% of PHB concentration. Stereolithography or an SLA 3D printing technique were used to evaluate the printability of the PHB/PUA blend resins. Additionally, from FESEM analysis, a change was observed in PUA’s microstructure, with an additional number of voids spotted. Furthermore, from XRD analysis, as PHB concentration increased, the crystallinity index (CI) also increased. This indicates the brittleness properties of the materials, which correlated to the weak performance of the tensile and impact properties. Next, the effect of PHB loading concentration within PHB/PUA blends and aging duration towards the mechanical performance of tensile and impact properties was also studied by using analysis of variance (ANOVA) with a two-way method. Finally, 12 wt.% of PHB/PUA was selected to 3D print the finger splint due to its characteristics, which are compatible to be used in finger bone fracture recovery.

## 1. Introduction

Recent development in the manufacturing industry has been a major important component to propel the Fourth Industrial Revolution (IR 4.0). Since its introduction in 2011, IR 4.0 has become the pioneer in the industrial movement on intelligent automation technology. The fundamental concepts are derived from several intelligent systems, which comprise the Internet of Things (IoT), big data, additive manufacturing, autonomous robotics, simulation, augmented/virtual reality (AR and VR), cloud computing and cyber security [1]. Among the intelligent systems, additive manufacturing is considered the new form of the smart factory, which is capable of achieving a reduction in manufacturing time, producing highly customizable complex designs and specific manufacturing designs based on individual customer’s needs, enabling faster procedures in prototype to final product decision making and promoting sustainability and resource efficiency [2].

Additive manufacturing, or as it is commonly called, 3D printing is an addition-based layering process of materials comprised of thermoplastic filaments, ceramics and metal utilizing 3D model objects constructed via computer-aided design (CAD) software. The advancement of this technology has been applied in many industries, such as aerospace, biomedical and automotive manufacturing [3]. There are various processes of additive manufacturing, which can be categorized as vat photopolymerization, sheet lamination, directed energy deposition, binder jetting, material jetting, powder bed fusion and material extrusion [4]. Each process differs from others in the aspect of principle and the materials used. Herein, the utilization of the stereolithography (SLA) method was derived from the vat photopolymerization process, which has the advantage of high-resolution printing, as well as the ability to produce an intricate design.

In the SLA method, the polymerization process occurs when the liquid resin is exposed to ultraviolet light energy to solidify the resin in forming a 3D structure [5]. The process starts with the laser illuminating a design at the surface of the resin and curing it according to the specific standard triangulated language (STL) file format. The platform then technically moves up and down from the resin’s vat/tank to allow the built-up for the next layer of the model structure. These stages continue until the specified model has been constructed into a solid 3D structure. Commonly, post curing treatment of the 3D structure is required to assure that all parts of the structure have been fully cured [6]. There are two fundamental components to practically operate the system, namely the types of laser beam and photopolymer used. Laser beams with a wavelength of 385 nm and 405 nm are required. A UV laser, in which the wavelength is shorter than 300 nm, is typically ineffective for SLA due to the absorption of its photon energy by the photoinitiator molecules of the resin [7]. Despite the importance of the laser’s wavelength, photopolymer resin is the most vital component, as not all polymeric materials are compatible with SLA. Particularly, the photopolymer resin of SLA necessitates having certain properties of low melting temperature (*T_m_*) and viscosity to keep the photopolymer in a liquidlike form throughout the process [8].

Photopolymer resin consists of three main elements, which are oligomers, photoinitiators and monomers. The integration of chemical reactions between monomers and oligomers via free radical system is the key factor in formulating photopolymers [9]. Acrylate and methacrylate are the common monomers used in microfabrication due to their easy processability. This has resulted in the further development of acrylate and methacrylate monomers to fulfill the demand for multifunctional materials. Several acrylate- and methacrylate-based resins that are available in the market are poly (ethylene glycol) diacrylate (PEGDA), urethane dimethacrylate (UDMA), triethylene glycol dimethacrylate (TEGDMA), bisphenol A-glycidyl methacrylate (Bis-GMA), trimethylolpropane triacrylate (TTA) and bisphenol A ethoxylate diacrylate (Bis-EDA) [10]. In spite of this, polyurethane acrylate (PUA) is one of the rising acrylate-based resins that are rapidly used nowadays, as other acrylic-based resins exhibit a high shrinkage rate during curing, poor mechanical performance and have higher viscosity [11].

Inherently, PUA is a unique class of urethane group, which is comprised of terminated urethane prepolymer derived from polyols with polyisocyanate that is capped together with acrylic functionality and vinyl monomers [12]. According to the previous study, PUA polymer recorded a good mechanical performance, with 72 MPa of tensile strength and 3.6 GPa of Young’s modulus [13]. PUA also possesses a few advantages compared with other UV-curable polymers, such as better adhesion to the substrates, good impact resistance, high flexibility, chemical and abrasion resistance and improved weatherability [14]. These features qualified PUA to be applied in various applications, especially in polymer concrete, as well as the electrical and medical field, respectively. However, most available UV-curable resins are petroleum-based, thus lacking in natural materials and sustainability characteristics, which can be found only in biopolymer, such as alginates, chitosan and polyhydroxbutyrate or PHB. 

Alginate is a type of a natural polymer commonly extracted from brown seaweed, while chitosan is derived from chitin by using the deacetylation process, which is considered as the second most abundant polysaccharide in nature [15,16]. Currently, both natural polymers were widely used in biomedical applications, especially in drug delivery systems and wound healing, due to their biodegradability and biocompatible properties, which adhere with the concepts of green environments and sustainability [17]. However, both alginate and chitosan have poor mechanical performance, as these natural polymer types consist of weak and flexible materials that restrict their use in load-bearing applications, especially in bone repair treatment [18,19]. In spite of the limitations of alginate and chitosan, PHB has become one of the most promising natural polymer replacements that can be applied in many biomedical devices due to its high mechanical properties, which are comparable to polypropylene [20]. 

PHB is a linear polyester derived from excess sources of carbon with the help of bacteria systems, such as Bacillus megaterium, Ralstonia eutropha and Cupriavidus metallidurans [21]. PHB is also identified to have a methyl functional group (CH_3_) and an ester linkage group (-COOR) in its chemical structure which are responsible for the thermoplasticity, crystallinity and hydrophobic properties of the polymer [22]. PHB is portrayed to have good mechanical characteristics with a 20–40 MPa range of tensile strength and a high recorded Young’s modulus between 3.0–3.5 GPa compared with other biopolymers [23]. Furthermore, PHB offers a lot of advantages over conventional polymers, such as biodegradability, biocompatibility and water-insoluble features that are suitable to be applied in the medical industry [24]. Despite all these advantages, PHB still has a drawback, which is high production costs compared with conventional polymer, thus limiting its full potential in producing medical devices. Therefore, the idea to blend PHB with PUA to complement each other by combining their respective advantages is considered an alternative way to overcome the above limitation. A PHB/PUA blend is expected to enable the production of low-cost bioplastic materials and the ability to tailor the mechanical properties of a material according to a specific application, especially in the medical field.

In this work, the research focuses on the blending of PHB and PUA as the composition of the material for additive manufacturing, which could be potentially used as a splint in finger bone fracture recovery. The adoption of a 3D-printed splint in patients’ recovery could address the limitations of conventional casting due to its lightweight properties, durability and ease of use. The printability of this new blend material was studied by using the SLA 3D printing technique. Next, the effect of PHB compositions within PUA on the morphology, structural properties, crystallinity, thermal stability, tensile properties and impact strength of the 3D-printed PHB/PUA samples was also studied. 

## 2. Experimental Materials and Methodology

### 2.1. Experimental Materials

Polyhydroxybutyrate (PHB) was supplied by Biomer Incorporation (Krailing, Germany) under the commercialized grade P309 and manufactured in powder form. The main properties of the polymer are average molecular weight (Mw) = 500–600 kDa and melt flow index (MFI) = 10 g/10 min (180 °C, 5 kg). 

Polyurethane acrylate (PUA), a photosensitive resin, was obtained from Shenzhen Anycubic Technology Co., Ltd. (Shenzen, China). According to the safety data sheet of the material (MSDS), it consists of around 30 to 60% PUA, a monomer which is acrylate between 10 to 40% of the PUA structure and is added with 25% of the photoinitiator element. 

Isopropanol (IPA) manufactured by Sigma-Aldrich (Burlington, MA, USA) was utilized during SLA postprocessing to remove the remaining residual of the blend resin that was left over after the printing process. The molecular weight (Mw) of IPA is 60.10 g/mol, while the density is 0.785 g/mL at 25 °C based on MSDS from Sigma-Aldrich.

### 2.2. Formulation of PHB/PUA Blend Resin Compositions

PHB powders were dried in a vacuum oven for 8 h at 80 °C to eliminate any moisture that was initially trapped in the molecule of PHB. An analytical weighing balance was used to weigh the PHB powders and PUA resin separately according to the selected proportions, as presented in Table 1 below. All PHB/PUA blend compositions were prepared by polymer blending method based on the various proportions of PHB weight percentage: 0, 6, 12 and 18 wt.%. 

Next, all the blend materials based on their composition were mixed in separate veils by the magnetic stirrer (WiseStir MSH-20D, Witeg, Germany). Opaque veils were used to prevent the exposure of the mixed composition to UV light. The PHB/PUA blend resin compositions were stirred at 250 rpm of rotational speed with 60 °C of processing temperature for 24 h. This process continued until each composition had reached its homogeneous level. After the mixing process, the blend solution was cooled down for 10 min to let it be equivalent to the ambient temperature before proceeding to the printing stage.

### 2.3. Three-Dimensional Model Design of the Samples

Initially, all the 3D-printed samples for each test were designed by using CAD software (Blender, Amsterdam, The Netherlands) according to the specific standards that were set up to assure the validity of the obtained results. The mechanical performances in this work were studied by sample with a dog-bone pattern of ASTM D638. Among the five types available in ASTM D638, Type V was selected with the measurements of 63.5 mm in length, 9.53 mm in width and 3.2 mm in height. The impact strength test required a rectangular shape of ASTM D256, with the same length and height as used in tensile measurement but different in width, which was 12.7 mm, with a V notched in the middle of the sample. 

Meanwhile, for the structural and thermal characteristics evaluation, 3D-printed samples of a circular pattern with a diameter of 8 mm and thickness 1.0 mm were constructed for Fourier transform infrared (FTIR) (Perkin Elmer, Waltham, MA, USA), field emission scanning electron microscopy (FESEM) (Hitachi SU8220, Tokyo, Japan) and thermogravimetric analysis (TGA) (TGA/SDTA851, Mettler Toledo Coro, Greifensee, Switzerland). X-ray diffraction evaluation (XRD) (Rigaku, Tokyo, Japan) used rectangular samples (19.5 mm × 19.5 mm × 0.5 mm). All the rendered designs were then transposed as STL files. These STL files finally generated viewable and readable data by a Photon S slicer and were ready to be used in the SLA 3D printer (Anycubic Photon S, Shenzhen, China).

### 2.4. Three-Dimensional Printing Process of PHB/PUA Blend Resin Compositions

The SLA 3D printer fabricated a triplicate of samples for each composition of the PHB/PUA blend, with 8 s of exposure time at once. Then, the 3D-printed samples underwent a post-3D-printing process to clean up all the remaining uncured solutions that were left around the sample’s area using isopropanol (IPA). All the samples went through an ultraviolet (UV)-cured process by using a curing machine (Form Cure, Formlabs, Somerville, MA, USA) for 50 min at 60 °C. This postcuring process is able to enhance the mechanical performance of the samples by strengthening the bond linkage of the resin blends.

**Figure 1:**
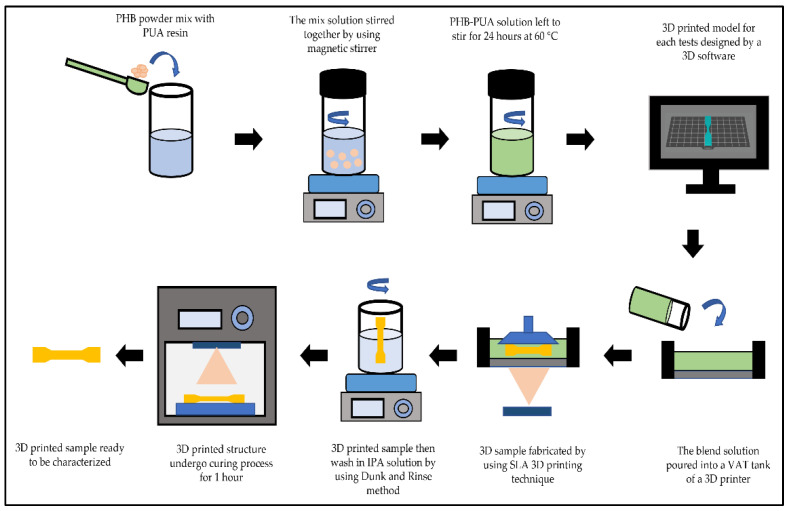
The overall procedure of 3D-printed PHB/PUA samples.

There was a total of 24 3D-printed samples for tensile and impact properties. The first 12 samples aged about one day in the desiccator, while the remaining 12 samples were left to age for a straight 30 days in the different desiccators. In the meantime, all the samples that were aged for a day were analyzed using FTIR, XRD, FESEM and TGA. The flow of the overall sample preparation process is portrayed in Figure 1 below. 

### 2.5. Three-Dimensional Printability of Pure PUA and PHB/PUA Blend Resin Compositions

A rheometer (DVNext Rheometer, AMETEK Brookfield, Middleborough, MA, USA) was utilized in this work to determine the viscosity value of the pure PUA resin and PHB/PUA blend composition. The reading for each sample composition was taken at room temperature, with the spindle’s (RV-04) rotation adjusted to 50 rpm.

### 2.6. Morphological Study of PHB/PUA Blend Resin Compositions

FESEM (Hitachi SU8220, Tokyo, Japan) was utilized to study the effect of different weight compositions of PHB on the surface morphology of PUA. All inspections and microphotographs of 3D-printed samples were obtained at 1K resolution. 

### 2.7. Infrared Sprectroscopy Analysis of PHB/PUA Blend Resin Compositions

The FTIR test was executed to identify the chemical bonding of PHB, PUA and 3D-printed PUA and PHB/PUA blend resin compositions. In this work, FTIR spectrometer with attenuated total reflectance (ATR) (Perkin Elmer, Waltham, MA, USA) was applied to study the structural properties of the samples, as it allows a direct FTIR measurement by using a 9 bounce of Diamond/ZnSe ATR crystal. 

A background spectrum needs to be measured before starting the new measurement for each sample. PHB powder, PUA-based resin and 3D-printed PUA and PHB/PUA blend compositions were measured by placing each of them on the surface of the ATR crystal. Then, a constant pressure was applied for each sample by using the ATR’s arm to achieve excellent interaction between analyte molecules and the crystal. The instrument’s resolution was 4 cm^−1^, and each measurement was the product of 25 scans in the midinfrared range within 400 cm^−1^–4000 cm^−1^. IPA solution was used to clean the ATR crystal gently before starting the new measurement.

### 2.8. Crystallinity Structure of PHB/PUA Blend Resin Compositions

XRD evaluation was carried out to figure out the crystalline peaks of pure PHB, PUA resin and 3D-printed PUA and PHB/PUA blend resin compositions by using an X-ray diffractometer (Rigaku, Tokyo, Japan). It was run at 30 kV and 10 mA, with radiation of CuKα 0.145 nm of wavelength. The scattering angle was adjusted from 2.0° to 35°; meanwhile, 10°s^−1^ of step duration was set up. 

Additionally, the average crystallite size of PHB powder was also determined according to the peak position and FWHM values from the XRD data of PHB. The obtained values are substituted into the Scherrer Equation (1) below, where *D* is the crystallite size (nm), *K* is the Scherrer constant (0.9), *λ* is the wavelength of the X-ray sources, *β* is the FWHM value in radians and θ is the peak positions.

(1) $$D=\frac{K\lambda }{\beta cos\theta }$$

OriginPro (OriginLab Corporation, Northampton, MA, USA) was used in determining the crystallinity index of the blend compositions according to the XRD data that were recorded, which correlated with the International Center for Diffraction Data (ICDD). Firstly, to measure the areas of crystalline peak, all XRD data need to undergo a baseline correction procedure. This step was important to ensure the accuracy of the position and intensity of the selected peaks. The selected peaks then go through integral calculation to obtain the area of crystalline peak. The same steps were used in measuring the area of peaks. The crystallinity index (CI) of each composition was calculated by inserting all the finding values into the Equation (2) below.

(2) $$\mathrm{Crystallinity}\;\mathrm{Index}\;\left(\mathrm{CI}\right)\%=\frac{\mathrm{Area}\;\mathrm{of}\;\mathrm{crystalline}\;\mathrm{peaks}}{\mathrm{Area}\;\mathrm{of}\;\mathrm{all}\;\mathrm{peaks}}\;\times 100$$

### 2.9. Thermal Analysis of PHB/PUA Blend Resin Compositions

Thermal properties of pure PHB, PUA resin, and 3D-printed PUA and PHB/PUA blend resin compositions were analyzed by a thermogravimetric instrument (TGA/SDTA851, Mettler Toledo Coro, Greifensee, Switzerland). This instrument measured the weight loss of the experimented materials with a specified range of temperatures. 

The measurement for this thermal analysis was performed in nitrogen N_2_ condition at 20 mL/min flow rate. Meanwhile, the heating scan rate of 20 °C/min was set up to control the temperature change in a range between 25 °C and 700 °C. 

### 2.10. Tensile Properties of PHB/PUA Blend Resin Compositions

Tensile measurement of all the samples was carried out using a Universal Materials Testing Machine (LR100K Lloyd Instrument, Hampshire, UK). The tests were run at ambient conditions with 5 mm/min of crosshead speed and 10 kN of the loaded cell. The stress–strain value of the tensile measurement was recorded by Bluehill universal testing software (Illinois ToolWorks Inc., Glenview, IL, USA).

### 2.11. Impact Properties of PHB/PUA Blend Resin Compositions

The V-notched rectangular specimens of ASTM D256 were used to study the impact performance of the PHB/PUA blend resin composition. This test was conducted by a Digital Impact Tester with Notcher (DG-IB, TokyoSeiki Co., Ltd., Otaku, Tokyo), which was set up at 11 J of nominal impact energy, and the rate of velocity impact was adjusted to 3.0 m/s.

### 2.12. Statistical Analysis of Mechanical Properties of PHB/PUA Blend Resin Compositions

For statistical analysis, the Statistical Package for the Social Sciences (SPSS v.21) (IBM Corporation, Armonk, NY, USA) was utilized to evaluate the mechanical results of the tensile and impact properties of the 3D-printed PHB/PUA blend compositions. Since the recorded data were in accordance with a normal distribution (*p* > 0.05), analysis of variance (ANOVA) with a two-way method was used to figure out the relationship between the various amount of PHB wt.% composition integrated into PUA resin and the duration of aging process of the 3D-printed PHB/PUA towards the mechanical performance of the specimens.

## 3. Results and Discussion

### 3.1. Parameters Affecting SLA 3D Printing

**Figure 2:**
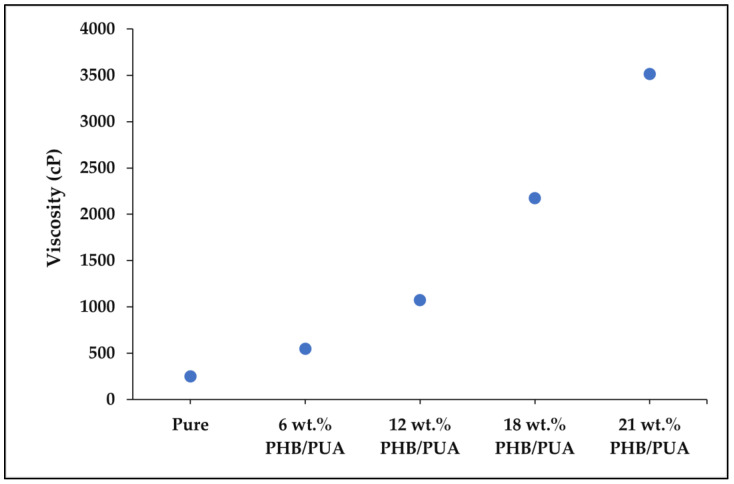
The viscosity value of PHB/PUA blends resin compositions.

Various factors could affect the printability of SLA 3D printers, such as material deformation, mechanical control, postprocessing time and spot diameter [25]. However, in SLA, viscosity is the most essential factor that could influence the whole structure of the system [26]. Each commercialized resin has its specific viscosity, as stated in the material datasheet (MDS) of the resin product. As PHB powders were integrated into the resin structure of PUA, it directly evolves the structural properties of the resin. The viscosity of PHB/PUA blend resin increased with the additional amount of PHB composition, as shown in Figure 2. As PHB content increases in the composition, it tends to cause the resin to agglomerate, which greatly increases the viscosity of the resin. 

Based on experiment that was carried out, it was observed that pure PUA resin obtained the viscosity value of 248 cP in ambient temperature, which is almost tailored with the information recorded in MDS whither between 150 and 250 cP. PHB/PUA blend resins with 18 wt.% of PHB content attained a value of around 2172 cP. It was the highest viscosity value for PHB/PUA blend resins that had successfully been printed. Meanwhile, for 21 wt.% of PHB concentration, it was recorded that the viscosity of the composition was too high, which was around 3512 cP. In certain cases, high viscosity can result in poor layering and voids where the resin fails to flow sufficiently into the areas required for the next layer. Since the resin needs to be able to flow back towards the vat tank’s center, it was recommended that the viscosities must be within a range which do not exceed the limit of their printability, which is between 200 cP and 2200 cP.

Furthermore, any changes that occur either in temperature or humidity throughout the SLA process will affect the printing outcomes [27]. Resins are photo inks that require as low viscosity as possible. It is important to maintain the resin warm at ambient temperature since the viscosity value decreases as temperature increases. Nonetheless, the resin’s temperature must not be too high to preserve the quality of the output product. The temperature of the material is optimized based on the material properties, as any alteration will affect the physical structure of the printed objects. Additionally, resin exposure time also had an impact on the 3D-printed structure [28]. Manufactured resins have a common standard of exposure time setting as well. However, as PHB powder was introduced to the resins, the standard exposure time setting was thrown off. The optimized printing parameters were discovered after several tests, as indicated in Table 2. 

### 3.2. Morphological Study

**Figure 3:**
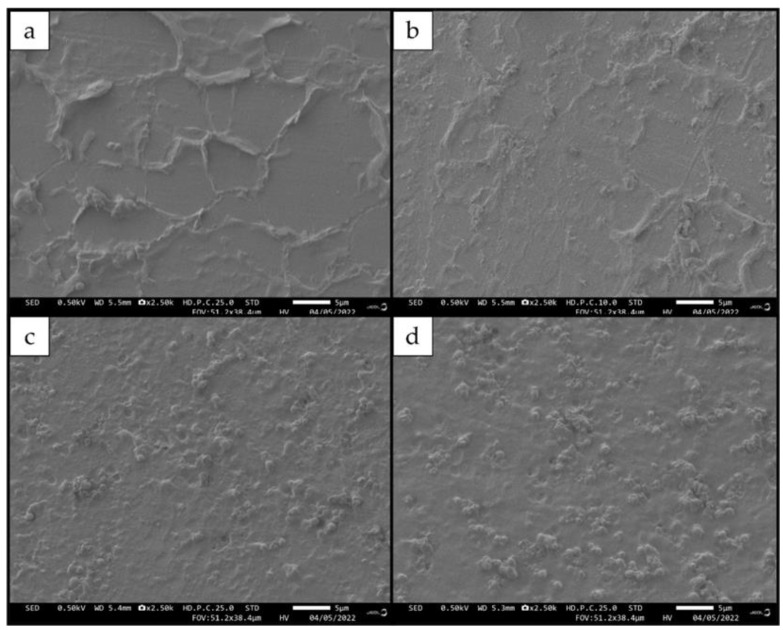
FESEM analysis of 3D-printed PHB/PUA samples: (**a**) PUA; (**b**) 6 wt.% PHB/PUA; (**c**) 12 wt.% PHB/PUA and (**d**) 18 wt.% PHB/PUA.

In this study, FESEM was used to examine the effect of PHB integration on the morphological structure of 3D-printed PHB/PUA blend compositions. Generally, FESEM helps in identifying the presence of voids on the particle’s surface structure, the homogeneity of the composite, the existence of particles agglomeration, and the potential orientation of the nanoparticles and their dispersion within the polymer matrix. Figure 3 portrays the micrographs of the 3D-printed films of PHB/PUA blend composite with various contents of PHB, which were (a) 0 wt.% PHB/PUA; (b) 6 wt.% PHB/PUA; (c) 12 wt.% PHB/PUA; and (d) 18 wt.% PHB/PUA. Based on Figure 3a, the surface structure of 3D-printed PUA is smooth with a laminar-like structure; however, it was observed that the integration of PHB powder into PUA matrix changed the microstructure of the film’s surface.

Based on past research, a good adhesion for filler–matrix is when there are no filler pullouts or gaps that emerge between the fillers and matrix during low loading of the fillers in the composite [29]. However, in Figure 3b, as the 6 wt.% of PHB was initially incorporated into the PUA matrix, it showed that some filler had pulled out from the matrix. It was observed that this phenomenon continued for 12 wt.% and 18 wt.% of PHB concentrations; as the amount of PHB powder increased, more agglomeration of particles occurred. This led to an increasing number of voids or microcracks that were visible on the surface structure of the 3D-printed film’s surface, as shown in Figure 3c,d. This happened due to the poor dispersion of PHB powders into the PUA matrix. Based on the observation of all these microphotographs, it can be concluded that the PHB powders are nanomaterials with an average particle diameter of less than 100 nm. Therefore, the findings were in line with the clustering impact of PHB particles as fillers that were affecting the mechanical properties of the PHB/PUA blend composite. The cluster effect refers to the tendency of the nanoparticles to accumulate and aggregate, thus producing a region with a high particle concentration in the matrix’s structure. This phenomenon will lead to a poor stress transfer within the matrix blend composite, thus resulting in a reduction in its tensile and impact strength [30]. 

### 3.3. Infrared Spectroscopy Analysis

**Figure 4:**
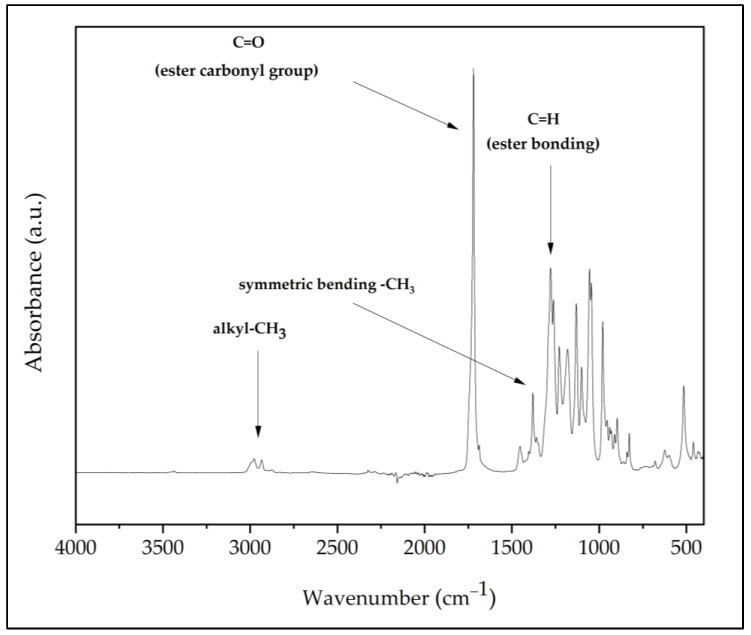
Infrared spectrum of PHB powder.

The infrared spectrum of the pure PHB powder displayed a strong absorption band at the 1721 cm^−1^ spectrum, which denotes the group of ester carbonyl that coincides with the C=O stretching bond located in the molecular network chain. Another absorption band was discovered at 1278 cm^−1^ and corresponded to the ester bonding group of C-H. Aside from that, a range of absorption bands were exhibited between 1163 cm^−1^ and 1210 cm^−1^, suggesting a stretching of the ester group’s C-O bond. Meanwhile, the bending vibrations at 2969 cm^−1^ and 2927 cm^−1^ indicated the existence of the methyl group, while at the same time, the peak located at 1377 cm^−1^ correlated to the symmetric bending of the methyl group. Furthermore, the band depicted at 1452 cm^−1^ indicated the asymmetric bending of –CH_2_ and –CH_3_. Lastly, a medium intense band at 3434 cm^−1^ was attributed to the group of hydroxyls. Figure 4 shows the infrared spectrum of PHB powder. 

**Figure 5:**
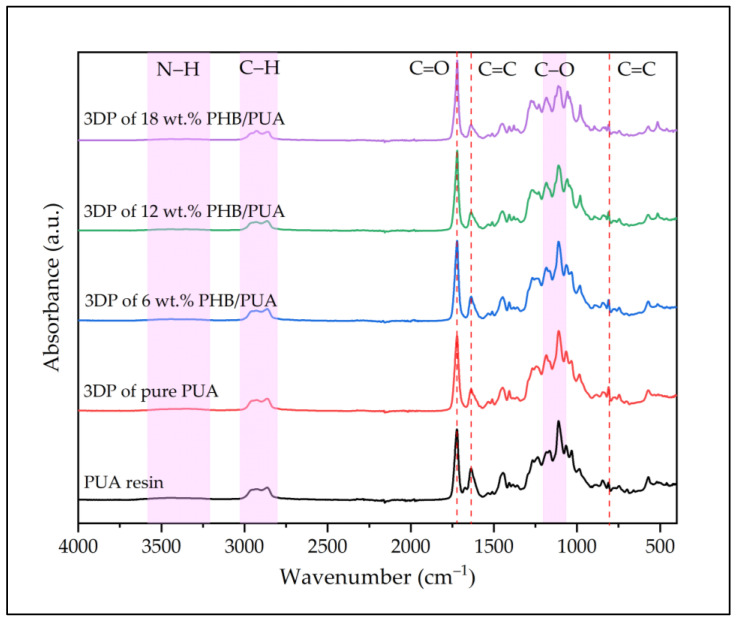
Infrared spectrum of PUA resin and 3D-printed PUA and PHB/PUA blend compositions.

Figure 5 presents the infrared spectrum of PUA resin and various 3D-printed PHB/PUA samples. In the PUA spectrum, the properties of the absorption band depicted between 3200 cm^−1^ and 3600 cm^−1^ referred to the absorption vibration stretch of N-H in the urethane structure, while the N-H bending spectrum was observed at 1644 cm^−1^. Additionally, a series of absorption bands between 2800 cm^−1^ and 3000 cm^−1^ was linked to the vibration stretch of the C-H group. Meanwhile, a sharp absorption peak was clearly depicted at 1718 cm^−1^ that approved the existence of the C=O group in PUA formulation. Furthermore, in the spectra of 1644 cm^−1^, a peak was formed before the UV curing, which correlated to the stretching bonds of C=C. This reveals that methacrylate groups were fully integrated into the polyurethane chains [31]. However, this peak slowly disappeared in the spectra after the UV-curing process, but another peak started to draw at 815 cm^−1^. The group of methylene in the methacrylate molecule was associated with this peak, verifying the UV-crosslinking reaction [32]. Upon the addition and increase in PHB content in the PHB/PUA blend composite, the absorption bands located at 1163 cm^−1^ and 1210 cm^−1^ represented a stretching of the ester group’s C-O bond, which was observed to gradually decreases with the increase in PHB concentration. This change caused the C=O peak at 1718 cm^−1^ to surpass it and become the highest, especially when the PHB amount was increased.

### 3.4. Crystallinity Index (CI)

**Figure 6:**
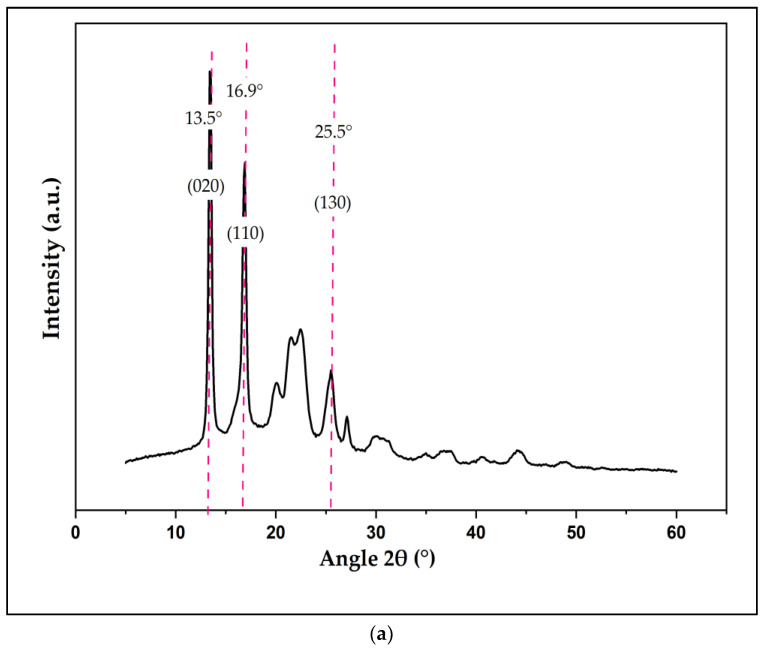

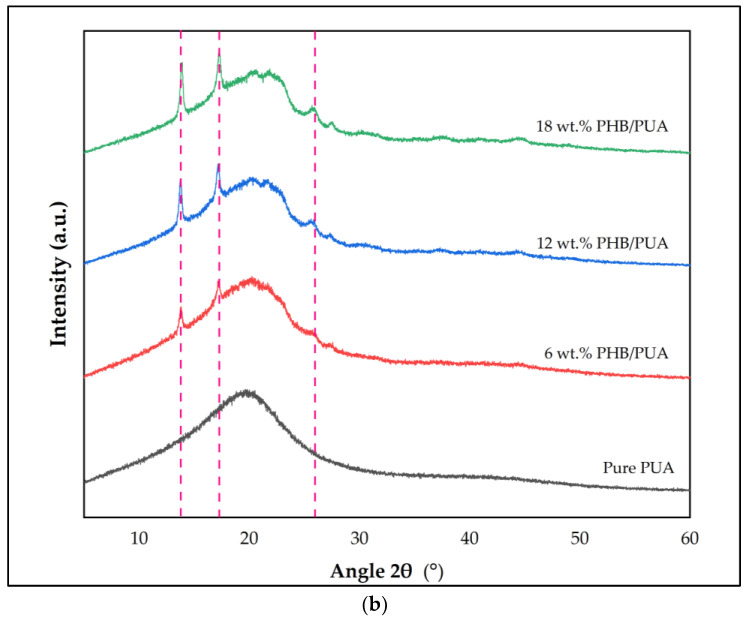
X-ray diffraction patterns: (**a**) PHB powder; (**b**) 3D-printed PUA and 3D-printed PHB/PUA.

In this study, XRD analysis was used to calculate the average crystallite diameter of the PHB powder. Based on the Scherrer equation, the average crystallite size of the PHB powder was around 10.74 nm. Table 3 below shows the full calculation results of the PHB’s crystallite size according to its peak position and full width at half maximum (FWHM) values. Other than that, XRD also examined the crystalline composition of the polymeric blend materials. The result of the XRD pattern of PHB powder and various 3D-printed PHB/PUA blend compositions is illustrated in Figure 6a,b below. As reported in our previous work, a 3D-printed amorphous PUA did not show any crystalline peak [33]. XRD analysis of the PHB powder also showed sequence peaks of crystalline, which was acknowledged by the Joint Committee on Powder Diffraction Standards (JCPDS) to have been discovered at the points of 13.5°, 16.9° and 25.5° [34]. PHB’s unit cell consists of an orthorhombic crystalline structure system. Furthermore, XRD patterns of 3D-printed PHB/PUA blend compositions depicted the same series of peaks as PHB powder, which were attributed as (020), (110) and (130), respectively. This confirmed that the level of crystallinity within 3D-printed PHB/PUA blend compositions was affected by the integration of PHB powder into the amorphous structure of the PUA resin. 

As the content of filler increases, more crystalline peaks appear in the PHB/PUA blend composites. When the PHB amount was increased in the blend compositions, the peaks located at the points of 13.5°, 16.9° and 25.5° started to appear and become sharper and thus corresponded with the hypothesis. The crystallinity index (CI) obtained for the powder of PHB was 91.52%, while 6 wt.% PHB/PUA recorded around 9.54% of CI. Consequently, among the various compositions of 3D-printed PHB/PUA, 18 wt.% of PHB concentrations were highlighted as the composition that displayed the highest CI, with a 22.05% crystallinity index value. This analysis resulted in demonstrating the brittleness region in 3D-printed PHB/PUA blend composites increased as the PHB’s high peaks became more visible when the amount of PHB loaded into the PUA structure incremented. Commonly, materials that obtain a high value of crystallinity index (CI) will also have a high-degree order of atom arrangement in their crystal lattice [35]. This will make the mobility of the atoms more restricted whenever the material is subjected to any stress, which eventually leads to poor energy absorption and thus causes the material to brittle and fracture easily [36]. Table 4 represents the crystallinity index for various compositions of 3D-printed PHB/PUA.

### 3.5. Thermal Properties

**Figure 7:**
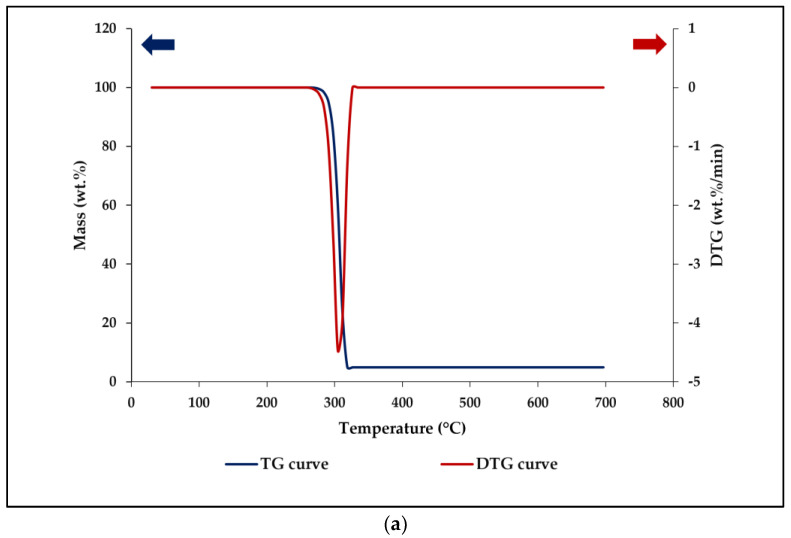

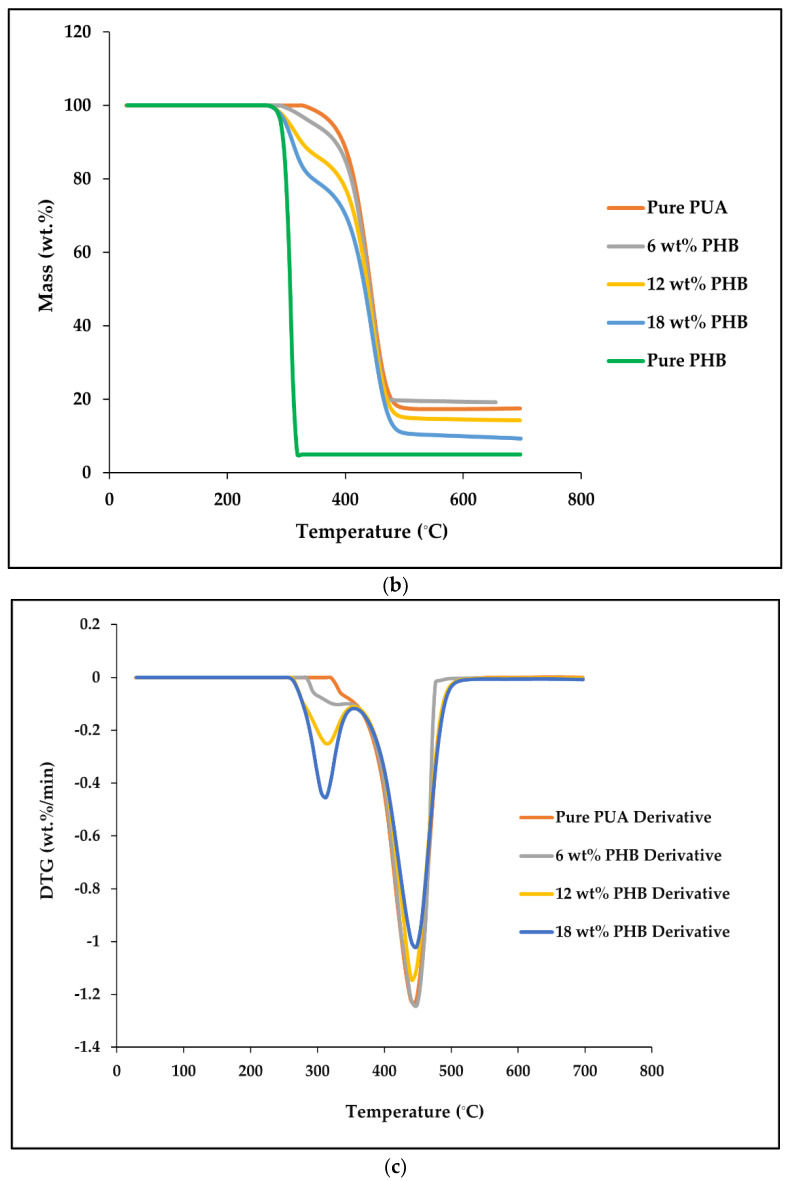
The thermal analysis of (**a**) TG curve and DTG curve of PHB powder; (**b**) TG curves of 3D-printed PHB/PUA blend composites; and (**c**) DTG curves of 3D-printed PHB/PUA blend composites.

It was discovered that 3D-printed PUA resin displayed only one step of the degradation profile. Based on the previous study, it was exhibited that the neat PUA resin had a single step of degradation profile whenever the temperature increased during the measurement [37]. Additionally, it was spotted that a single step of thermal degradation took place for the pure PHB powder, which was starting at the temperature of 262 °C and ended up at 326 °C, with a 95.06% weight reduction. The powder of PHB displayed the highest degradation peak, which was located at 305 °C, as shown in Figure 7a.

All TGA curves of different 3D-printed PHB/PUA compositions are shown in Figure 7b. The 3D-printed PUA exhibited a single step of thermal degradation profile that began at 355 °C and ended at 503 °C, leaving 17.45% of residue weight loss. The maximal degradation peak for 3D-printed PUA took place at 440 °C. This degradation was related to the breakage of chemical bonds for both urethane and ether groups during the decomposition process [38].

Figure 7c illustrates the additional number of peaks for 3D-printed PHB/PUA that started to turn up after the amount of PHB was increased for each composition. Based on the graph from Figure 7c, 3D-printed PHB/PUA was comprised of two steps of degradation profile. It was observed that as the amount of PHB was increased; the first maximal degradation peak for each 3D-printed PHB/PUA will begin to shift towards a lower temperature. This indicated that the material was becoming slightly reduced in its thermal stability but still well above the temperature of common materials used in fabricating a finger splint, as the first maximal degradation peak for each 3D-printed PHB/PUA composition was recorded at 325 °C, 320 °C and 313 °C for 6, 12 and 18 wt.% [39]. This occurred either due to the change in chemical composition of the material, the modification in crystalline structure or the presence of additives in a blend composite, which could be a factor that affects the thermal stability of the material [40]. 

Other than that, the thermal stability of a polymer also could be determined by the residual weight of the TGA samples. Based on the results in Table 5 below, 3D-printed PHB/PUA compositions recorded a reduction in their residual weight, which was 19.67%, 14.85% and 10.56% for each of the 6, 12 and 18 wt.% blend compositions. These results present that the thermal stability of the blend compositions started to decrease, as thermal stability is the ability of a material to withstand the effect of heat that could affect the material’s physical properties [41]. Table 5 below shows the thermal decompositions of the blend compositions and their correlation toward weight reduction (%) of samples. 

### 3.6. Tensile Properties

In this study, the Shapiro–Wilk test was used to determine factors that control the tensile properties of the PHB/PUA blend polymer. The factors include the various amount of PHB powder (wt.%) integrated into the PUA resin structure and the duration of the aging process for PHB/PUA 3D-printed samples. The result from this test shows that the tensile properties for all blend compositions of PHB/PUA are distributed normally at a significant value of *p* > 0.05. A two-way ANOVA analysis method was applied to examine the influence of PHB amount in the PHB/PUA blend compositions and the duration set for the aging process on the tensile properties of all the samples. The effect of the amount of PHB loaded (wt.%) into the PUA structure, duration taken for aging process and the relationship between both parameters are labeled as A, B and A × B, respectively. Table 6 shows the result of the influencing factors of those parameters on Young’s modulus, tensile stress and tensile strain that was systematically investigated, as the values of F and *p* are essential to acknowledge the null hypotheses. 

The hypotheses are neglected if the resulting value of F in Table 6 is higher than the F-value that was set by the critical value of F in the F-table [42]. Meanwhile, the *p*-value verified the reliability of the null hypothesis. In this work, for example, when the effect of the amount of PHB (wt.%) loaded into PUA structure towards Young’s modulus was analyzed, degrees of freedom (DF) in the numerator was determined as 3, while the DF for the denominator resulted at a value of 8. According to the F-table, the critical value was fixed at 4.07. The value of F presented in Table 3 (=7.34) is higher than the value in the F-table (=4.07) at α = 0.05. In short, the null hypotheses will be neglected. The F and *p*-value for the other parameters towards tensile properties (Young’s modulus, tensile strength and tensile strain) were obtained by applying the same method of analysis. Table 3 states that the duration of the aging process and the relationship between both main parameters were not statistically different, as the *p*-values were more than 0.05. 

**Figure 8:**
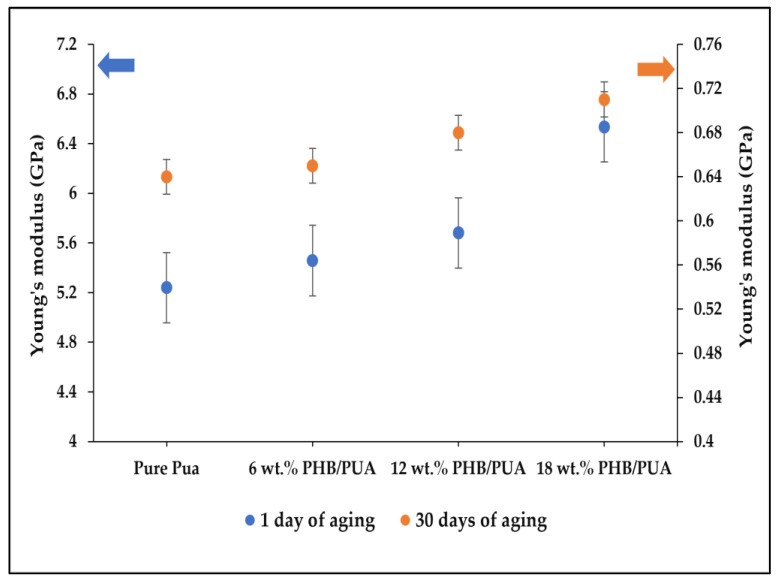
Young’s modulus of various 3D-printed PHB/PUA compositions at different aging durations.

Figure 8 presents the result of Young’s modulus with various blend compositions of 3D-printed PHB/PUA at different aging durations. It can be seen that from the graph, there was an increment in Young’s modulus value as the amount of PHB loading was increased, and a maximum value of 6.53 GPa was obtained at 18 wt% of loaded PHB powder. The significant improvement in PHB/PUA’s Young’s modulus is due to the capability of PHB to retain the applied stress and restrain the transfer of molecular chains in the PUA matrix whenever a load is applied to the composites. Additionally, the results are in line with the experimental data, which stated that the sufficient stress transfer of filler within the PUA matrix could lead to the increase in modulus [43]. Other than that, a high Young’s modulus indicates the stiffness of a material; so, based on the result above, it was corelated that PHB is one type of semicrystalline polymer that could contribute to the improvement of stiffness for the blend of PHB/PUA composites.

**Figure 9:**
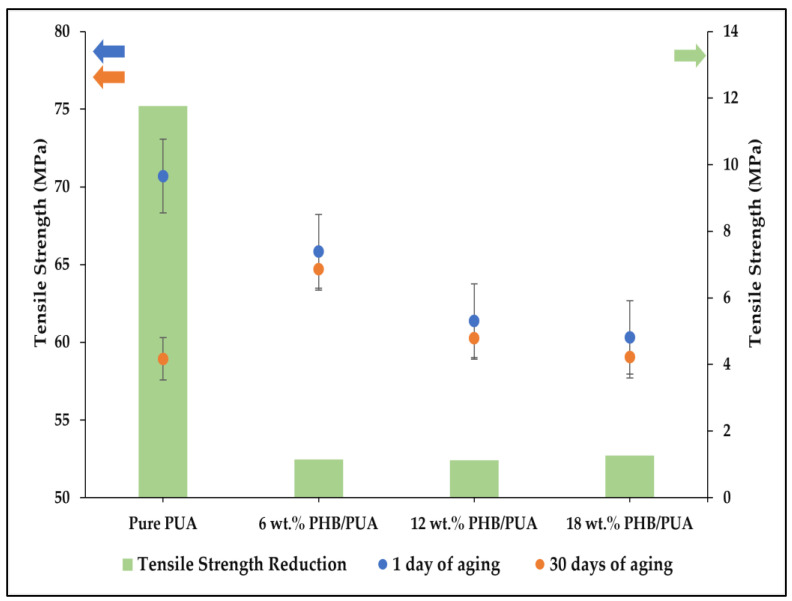
Tensile strength of various 3D-printed PHB/PUA compositions at different aging durations.

Figure 9 illustrates the results of tensile strength for the 3D-printed PHB/PUA blend composition at different aging durations. It could be explained from this figure that the tensile strength performance decreased with the increased amount of PHB loaded into the blend composition. The average tensile strength was 70.69 MPa at 0 wt.% PHB loading, but it reduced to 65.84 MPa for the 6 wt.% of filler loading. Tensile strength for the remaining compositions kept decreasing for 12 wt.% and 18 wt.% of PHB compositions; the tensile strength recorded was 61.38 MPa and 60.31 MPa each. This happened due to the agglomeration phenomenon that occurred during the blending of the composites, which denotes the inefficiency of the stress transfer behavior as the percentage of filler loaded into the blend compositions escalated [44]. Therefore, a local stress concentration will be initiated when a force has been applied to the blend composite materials subsequent to poor stress distribution in the composite’s structure [45]. Thus, some cracks started to develop on each stressed area of the matrix–filler linkage [46]. 

Despite this, the integration of PHB into the structure of the PUA matrix successfully retained the tensile strength value of the blend composites. Based on the change in tensile strength as shown in Figure 9, there was a huge loss of strength for pure PUA after a month of aging, with about 12 MPa of reduction value. However, the addition of PHB into the blend composites did not show any drastic loss of tensile strength value after a month of aging. Moreover, each composition of the PHB/PUA blend only recorded a decrease of below 2% compared with its initial tensile strength, which shows the capability of PHB to retain the strength of other polymer matrices.

**Figure 10:**
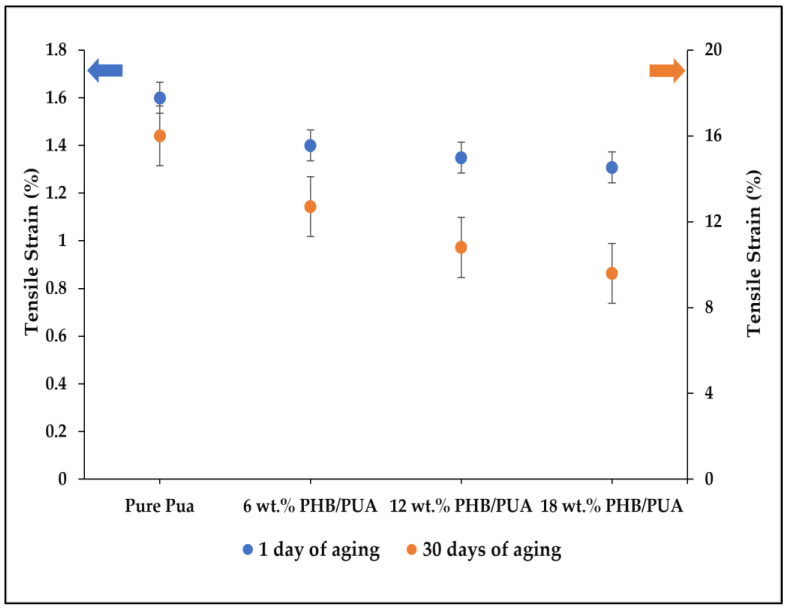
Tensile strain of various 3D-printed PHB/PUA compositions at different aging durations.

The tensile strain of various 3D-printed PHB/PUA compositions at different aging durations is recorded and illustrated in Figure 10. From the tensile strain result, it can be stated that the tensile strain slowly started to decrease with the increase in PHB powder content in the PHB/PUA blend compositions. The 18 wt.% of PHB/PUA blend recorded the lowest value of tensile strain at 1.31%, while the highest value of strain owned by 3D-printed pure PUA was 1.60%. The reduction in tensile strain occurred due to the low elongation of the filler, which later limited the elongation of the polymer blend matrix [47]. Consequently, the filler inhibited the polymer matrix’s capability to deform more than the composites’ overall deformation, thus resulting in a composite that is stiff but poor in ductility [48].

### 3.7. Impact Strength

As for the impact strength statistical analysis, the two-way ANOVA method was applied since there was no proof of non-normality towards impact strength for all compositions of 3D-printed PHB/PUA blend composites according to the Shapiro–Wilk evaluation. This analysis was carried out to investigate the influence of parameters, which were various loadings of PHB powder (wt.%) integrated into PUA resin structure and the duration of the aging process for PHB/PUA 3D-printed samples. The statistical analysis results of the impact strength are shown in Table 7. The weight percentage of PHB in the PHB/PUA blend composition has a significant effect on the impact strength, where 0.001 of *p*-value was recorded, which is lower than 0.05. In fact, from the table, it was found that the duration of the aging process and the relationship between both main parameters were not statistically different, as the *p*-values were more than 0.05.

**Figure 11:**
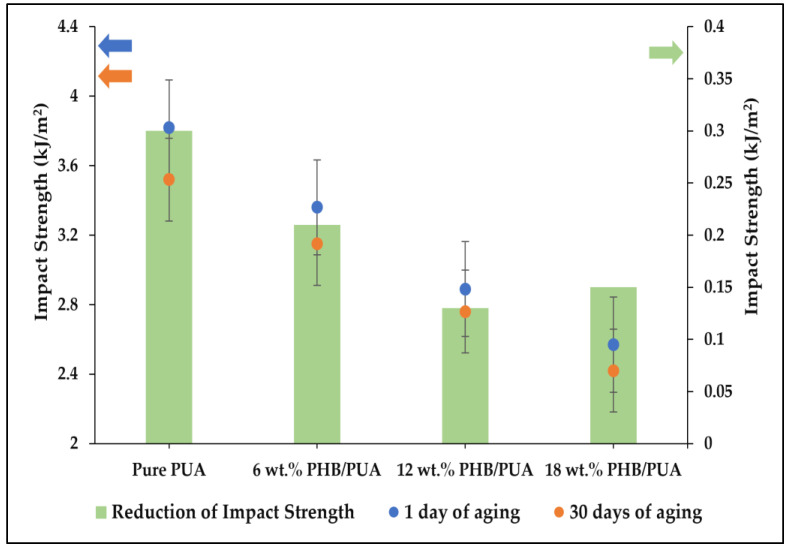
Impact strength value of various 3D-printed PHB/PUA compositions at different aging durations.

The impact properties of PUA and PHB blend composites are summarized in Figure 11 below. Pure PUA recorded the highest toughness, with an average of 3.82 kJ/m^2^, while 18 wt.% of blend composition between PUA and PHB recorded the lowest toughness on its impact properties, with an average of 2.57 kJ/m^2^. The rest of the blend compositions of PUA/PHB recorded an average impact strength of 3.36 kJ/m^2^ for 6 wt.% and 2.89 kJ/m^2^ for 12 wt.% blend compositions. Furthermore, the impact strength results of the varied 3D-printed PHB/PUA samples after aging for a month show the same type of decreasing pattern as the 3D-printed samples of one-day aging. From this graph, it was found that the impact strength of the PHB/PUA blend composite decreased with the increase in PHB composition, and further decreases can be observed when the composites aged for 30 days.

It was reported in the previous literature that impact strength is affected by crack propagation and the energy-absorbing capacity of the blend polymer. Crack propagation was a measure of the resistance of a material to the propagation of an existing crack, and sometimes it is called the fracture toughness of a material [49]. Crack propagation usually starts from a single point of failure, which is also known as a crack tip inside the material. Then, from that, the crack tip propagates and grows throughout the material, where more ripples form at this stage before the fracture occurs. However, the crack tip depends on the type of the material, either crystalline, semicrystalline, or amorphous, as different materials result in different mechanical properties. Since PHB is a semicrystalline polymer, the increase in PHB concentration led to the poor energy-absorbing capacity of the blend composite prior to fracture [50]. The sharpened crack tip of the crystalline material easily allowed crack propagation compared with an amorphous material, due to the ordered lattice structure of the crystalline region in the blend composite, which resisted any deformation when a force was applied, thus increasing the stress concentration at the crack tip [51].

### 3.8. Application in Additive Manufacturing Applications

The PHB/PUA blend composite can be a potential candidate material as a 3D-printed solution in medical applications; for example, in the case of a broken finger, a better finger splint is needed. First and foremost, the broken finger of the model patient will undergo 3D scanning by using a portable 3D scanner (3D Systems, Rock Hill, SC, USA) to acquire the SLA file of the broken finger. The 3D subject was scanned with a 360° view to make sure the outcome product fit well with the model patient. The scanning process took about 3–5 min to be completed before the 3D model could be projected into the 3D modeling software (Blender, Amsterdam, The Netherlands) and later converted as a standard tessellation language (STL) file.

In the second step, the 3D model of the broken finger will go through further refinement and reconstruction in certain areas that were unable to have a proper scan during the 3D scanning process. Then, by utilizing the same 3D software, a 3D model design of the finger splint was generated based on the previous STL file. The 3D model of the finger splint cast was designed with a circular pattern to make sure the circulation of air will occur between the 3D-printed splint cast and the finger’s skin. It is reported that air circulation is important to avoid moisture retention on the finger’s skin and the development of odors [52].

Next, the 3D model of the finger splint will be 3D-printed by using the 12 wt.% of PHB/PUA blend resin, since its characteristics were tallied with the properties needed for a broken finger recovery. A finger splint with a recovery purpose must be made up of materials that balance its stiffness and flexibility, not just providing adequate support to the injured finger but at the same time allowing some minor movement so that rehabilitation can take place along the recovery process. According to the tensile evaluation of the blend compositions, 6 wt.% recorded the highest value of tensile strain at 1.4%, while 18 wt.% recorded the highest value of Young’s modulus at 6.53 GPa. In producing a finger splint with a recovery purpose, the finger splint must not obtain a high value of Young’s modulus and tensile strain. A high Young’s modulus will cause the finger splint to become too stiff, which restricts the rehabilitation process and leads to poor healing. Meanwhile, if the finger splint is too flexible, it may not provide enough support and thus not immobilize the recovery finger effectively. Based on this evaluation, 12 wt.% was selected, as it recorded a moderate value of stiffness and flexibility among other compositions, with 5.68 GPa of Young’s modulus and 1.35% tensile strain. 

**Figure 12:**
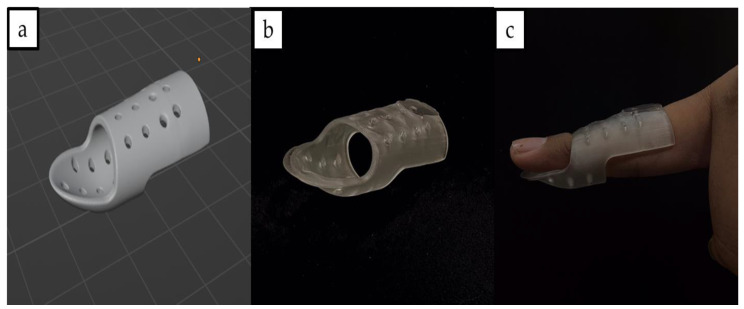
(**a**) Three-dimensional model design of splint; (**b**) 3D-printed splint; (**c**) 3D-printed split fitted well with the model’s finger.

Lastly, to ensure that a high-quality splint could be fabricated, the exposure time of the printer was set at 8 s, as it was the optimal setting according to the prior assessment. Additionally, around 15 g of the 12 wt.% of PHB/PUA blend resins were needed to fabricate the 3D-printed finger splint cast. During the post-printing procedure, IPA was used to clean the 3D-printed splint cast so that no resin residue was left over before undergoing the curing process. Finally, the 3D-printed splint cast was cured by using a UV-curing machine for 50 min at 60 °C. Based on the previous study in the other literature, the application of the traditional plaster of Paris casting on patients eventually brought them to a discomfort situation, as traditional plaster casting is impossible to adjust once it has hardened, and it sometimes does not fit properly with the patient’s fractured bone area [53]. Therefore, the use of 3D-printed splints as an alternative option for patients’ treatment could overcome the limitations of traditional casting, as it is light in weight, durable and easy to use. The 3D model of the splint and its product are shown in Figure 12a and Figure 12b, respectively. Meanwhile, Figure 12c presents the wearing of the 3D-printed splint on the model’s finger.

## 4. Conclusions

This research concentrates on the mechanical and structural properties of PHB/PUA blend compositions as an advanced material that can be applied in the healthcare industry. The blend of PUA resin with PHB powder was formulated, and 18 wt.% of PHB is the optimal amount that could be incorporated into the PUA matrix with a 2172 cP viscosity value. In fact, it was the optimum point of viscosity of the PHB/PUA blend composites that were successfully printed. The maximum amount that was formulated was 21 wt.% of PHB/PUA; however, the viscosity recorded for this composition was too high, as 3512 cP of viscosity value was obtained. High viscosity can result in poor layering and voids where the resin fails to flow sufficiently into the areas required for the next layer. Next, from surface morphology, it was observed that the addition of PHB powder into the PUA matrix changes its microstructure’s surface. The additional number of voids or microcracks started to become visible on the 3D-printed films’ surface, which may be caused by the uneven dispersion of PHB into the PUA matrix. Then, for FTIR analysis, a chemical chain reaction took place at the double bonds of methacrylates or acrylates, suggesting that the double bonds reacted with the free radicals generated by the photoinitiator. This indicated that the materials were well exposed to UV light throughout the 3D printing process. 

Furthermore, the number of the crystallinity index (CI) in XRD measurement increased as the PHB content in PHB/PUA blend compositions increased. A total of 18 wt.% of PHB/PUA blend resin recorded the highest crystallinity measurement, with 22.05% crystallinity index (CI). Materials with a high value of crystallinity index (CI) will have a high-degree order of atom arrangement in their crystal lattice. Thus, this restricts the mobility of the atoms when a force is applied, resulting in the material being brittle and leading to fracture. Additionally, as expected in TGA analysis, neat PUA resin only had a single-step degradation profile, while PHB/PUA blend resins were recognized to have a two-step degradation profile. Moreover, based on statistical analysis, the amount of PHB content in the PHB/PUA blend composition has a significant effect on the tensile and impact strength, where the *p*-value recorded was lower than 0.05. The clustering effect between nanoparticles of PHB in the polymer matrix is the main factor that leads to the reduction in mechanical performance. This happened due to the inefficient stress transfer behavior that occurred inside the composite. Finally, a successful 3D-printed finger splint based on a 12 wt.% PHB/PUA blend resin was constructed. Moreover, the composition was selected due to its characteristics, which were moderate in stiffness and flexibility and suitable for a finger splint with a recovery purpose.

## Figures and Tables

**Table 1:** Different compositions of PHB incorporated into PUA-based resin.

Composition (%)	PHB Powder (g)	PUA Resin (g)
Pure PUA	0	10
6 wt.% PHB/PUA	0.64	10
12 wt.% PHB/PUA	1.36	10
18 wt.% PHB/PUA	2.20	10

**Table 2:** The optimized printing setting for PHB/PUA blends resin.

Specifications	Value
Layer of the thickness (mm)	0.05 mm
Time exposure at normal (s)	8 s
Off time (s)	1 s
Time exposure at bottom (s)	60 s
Number of layers at bottom	3

**Table 3:** Crystallite size for PHB powder according to peak position and FWHM values.

Peak Position (Radians)	FWHM (Radians)	Crystallite Size (nm)
13.44	0.56	14.36
16.83	0.66	12.25
25.44	0.77	10.54
20.25	17.16	0.47
27.10	0.36	23.01
21.93	2.11	3.83
Average Crystallite Size (nm)		10.74

**Table 4:** Crystallinity Index (CI) for PHB powder and 3D-printed PHB/PUA.

Weight Composition (%)	Crystallinity Index (%)
PHB powder	91.52
6 wt.% PHB/PUA	9.54
12 wt.% PHB/PUA	16.27
18 wt.% PHB/PUA	22.05

**Table 5:** Thermal decompositions and their correlation towards weight reduction (%) of samples.

Samples	PHB Powder	Pure PUA	6 wt.% PHB/PUA	12 wt.% PHB/PUA	18 wt.% PHB/PUA
T_1_ (°C)	262–326	355–503	282–354	263–356	255–356
T_2_ (°C)	-	-	354–496	356–510	356–504
Residual Weight	4.95	17.45	19.67	14.85	10.56

**Table 6:** Statistical analysis results of various 3D-printed PHB/PUA compositions on tensile characteristics.

Two-Way Anova	Young’s Modulus			Tensile Strength			Tensile Strain		
	A	B	A × B	A	B	A × B	A	B	A × B
Degree of Freedom	3	1	3	3	1	3	3	1	3
Sum of Squares	1.580	153.824	1.238	453.416	4.217	1.301	97.747	815.500	87.731
Mean Squares	0.527	153.824	0.413	151.139	4.217	0.434	32.582	815.500	29.244
F-value	9.728	2841.359	7.623	24.081	0.672	0.069	10.377	259.716	9.313
*p*-value	0.001	0.000	0.002	0.000	0.424	0.976	0.000	0.000	0.001

**Table 7:** Statistical analysis results of various 3D-printed PHB/PUA compositions on impact characteristics.

Two Way Anova	Impact Strength		
	A	B	A × B
Degree of Freedom	3	1	3
Sum of Squares	5.126	0.182	0.006
Mean Squares	1.709	0.182	0.002
F-value	31.424	3.347	0.037
*p*-value	0.000	0.086	0.990

## Data Availability

Not applicable.
